# Historical phenotypic data from seven decades of seed regeneration in a wheat *ex situ* collection

**DOI:** 10.1038/s41597-019-0146-y

**Published:** 2019-07-29

**Authors:** Norman Philipp, Stephan Weise, Markus Oppermann, Andreas Börner, Jens Keilwagen, Benjamin Kilian, Daniel Arend, Yusheng Zhao, Andreas Graner, Jochen C. Reif, Albert W. Schulthess

**Affiliations:** 1 Department of Breeding Research, Leibniz Institute of Plant Genetics and Crop Plant Research (IPK), Corrensstr. 3, 06466 Seeland/OT, Gatersleben, Germany; 2 Department of Genebank, Leibniz Institute of Plant Genetics and Crop Plant Research (IPK), Corrensstr. 3, 06466 Seeland/OT, Gatersleben, Germany; 3 Institute for Biosafety in Plant Biotechnology, Julius Kühn-Institut (JKI) – Federal Research Centre for Cultivated Plants, 06484 Quedlinburg, Germany; 4 Global Crop Diversity Trust, Platz der Vereinten Nationen 7, 53113 Bonn, Germany

**Keywords:** Plant breeding, Data publication and archiving, Statistical methods, Data processing, Natural variation in plants

## Abstract

Genebanks are valuable sources of genetic diversity, which can help to cope with future problems of global food security caused by a continuously growing population, stagnating yields and climate change. However, the scarcity of phenotypic and genotypic characterization of genebank accessions severely restricts their use in plant breeding. To warrant the seed integrity of individual accessions during periodical regeneration cycles in the field phenotypic characterizations are performed. This study provides non-orthogonal historical data of 12,754 spring and winter wheat accessions characterized for flowering time, plant height, and thousand grain weight during 70 years of seed regeneration at the German genebank. Supported by historical weather observations outliers were removed following a previously described quality assessment pipeline. In this way, ready-to-use processed phenotypic data across regeneration years were generated and further validated. We encourage international and national genebanks to increase their efforts to transform into bio-digital resource centers. A first important step could consist in unlocking their historical data treasures that allows an educated choice of accessions by scientists and breeders.

## Abstract (summary)

**Table:** 

Design Type(s)	data integration objective • data validation objective • data cleaning objective
Measurement Type(s)	databank_entry
Technology Type(s)	digital curation
Factor Type(s)	Season • temporal_instant • temporal_interval • geographic location
Sample Characteristic(s)	Triticum aestivum • seed • cropland biome

Machine-accessible metadata file describing the reported data (ISA-Tab format)

## Background & Summary

The global cereal production amounted to 2.65 billion tons during the 2016–2017 growing season, with maize, wheat, and rice being the most relevant crops, accounting for 41.16%, 28.55%, and 19.04%, respectively^1^. Cereals are used as food and feed^2^. For feeding 9 billion people by the year 2050 food production must be doubled corresponding to an annual growth rate of 2.4%^3^. However, cereal production was merely increased by 1.46% during the last growing season^1^ and the current global annual wheat yield progress of 0.9% will be also insufficient to meet the future human needs^3^. Moreover, in some important wheat growing regions of Europe the yield progress is even stagnated since almost 20 years^4^. Additionally, limited arable land, the urgent need for more sustainable agriculture, the loss of biodiversity, changing socio-economic conditions in developing countries along with more-often occurring abiotic and biotic stress conditions due the climate change, pose further challenges for food production and food security^5^. In order to adapt wheat to these future demands, the use of genetic variation harbored within plant genetic resource collections has been frequently suggested^6–8^. Nevertheless, prior to the use of genetic resources for breeding, accessions with beneficial traits have to be identified^9^. This is not a trivial task if it is considered that around 856,168 wheat accessions are hosted in genebanks world-wide^10^. Therefore, the current scenario with insufficient genetic and phenotypic information imposes a major, if not the major, obstacle for the use of this extant genetic variation^6^.

The Federal *ex situ* Genebank of Agricultural and Horticultural Crops hosted by the Leibniz Institute of Plant Genetics and Crop Plant Research (IPK) in Gatersleben (Germany) ranks among the ten largest collections worldwide^11^. Put into numbers, this collection preserves ~151.000 accessions that comprise ~3.000 plant species of 756 genera^12^. Consequently, maintaining this huge genetic diversity requires the annual regeneration of about 5% of the collection. Moreover, the regeneration process is accompanied by routine phenotypic characterizations, which are required to preserve the seed integrity across regeneration cycles^13^. Nonetheless, harnessing this historical phenotypic data for breeding and research is challenging and several strategies have been described for analyzing and validating such non-orthogonal datasets^12,14,15^.

Following the FAIR principles^16^, this study provides original historical data of 12,754 spring and winter wheat accessions characterized for flowering time (FT), plant height (PH), and thousand grain weight (TGW) during 70 years of seed regeneration. Additionally, by applying a previously described quality assessment and analysis strategy^12^, we provide an outlier corrected dataset as well as ready-to-use processed phenotypic data in the form of Best Linear Unbiased Estimations (BLUEs) to allow for the direct comparison of accessions across regeneration years. Next to a standard outlier correction method, historical monthly weather data from the local weather station were used to remove outlier years and multi-environmental orthogonal field trials with random sampled accessions confirmed the high data quality.

The original historical phenotypic data can be reused to develop other outlier correction methods for highly non-orthogonal datasets different from the one presented here or to investigate the influence of weather parameters on the phenotypic performance. In contrast, the BLUEs will complement the passport information stored for each accession in the Genebank Information System (GBIS)^11^ and enable genebank users to have a more informed choice of accessions, e.g. for breeding and research purposes. Together with the recently published historical phenotypic barley (*Hordeum* sp.) data^17^ comprising 12,865 spring and winter accessions, phenotypic data is now available for approximately 60% and 64% of the IPK’s barley and bread wheat (*Triticum aestivum* L.) collections, respectively. Hence, this publication represents another important step in charging genebanks with phenotypic information and extending them to bio-digital resource centers. With the attached simplified software scripts for outlier correction and estimation of BLUEs, we want to encourage other genebanks to take advantage of their historical data treasures across crops and traits.

## Methods

### Plant material

The IPK wheat collection comprises more than 27,000 accessions of the genus *Triticum*, whereby the majority with about 20,000 accessions corresponds to the species *Triticum aestivum* L. More than 135 global collection expeditions, in addition to seed exchanges with other institutes as well as seed donations made this germplasm collection possible. Accession-relevant information such as taxonomic classification, accession name, collection site, and growth habit are documented as passport information in the Genebank Information System of the IPK (GBIS)^11^. Please note that the content of the passport information is under continuous improvement through ongoing research and data maintenance. The passport information used in this publication was retrieved on 12^th^ December 2016. In this study we focus exclusively on the species *Triticum aestivum* L., of which phenotypic data for FT, PH, and TGW were available for 12,754 accessions.

### Weather data

Since January 1953 consecutive monthly records for the average air temperature (T.avg), minimum air temperature (T.min) and maximum air temperature (T.max) measured two meters above ground in °C as well as precipitation (Rain) in mm and air humidity (Moisture) in % were available from the weather station located at the IPK campus (Gatersleben, Germany, latitude: 51°49′22.5′′N, longitude: 11°16′40.6′′E, 110.5 m.a.s.l.). Until 1992 the measures from the weather station were documented daily on a handwritten basis. This was replaced by electronic recording and manual chip card readout during the 1992–1996 period. Between 1996 and 2008 an external computer next to the station was automatically recording all parameters and since 2008 weather data were directly transferred to the central database management system of IPK. For January and February 1980 no weather data were available and records for T.min and T.max were missing between April 1993 and November 1999 as well as for the January-May period in 2007 and for July 2008 due to technical reasons. Until March 2001, the calculation of the average temperatures followed the approach of the so-called Mannheimer Stunden (four values per day). Since 2001, the averages have been calculated on 24 values per day.

### Phenotyping routine during seed regeneration as source of historical data

A central task of genebank management is the regeneration of the stored material. This becomes necessary when (i) seed stocks drop below a certain threshold, (ii) the germination rate has decreased below a critical threshold, (iii) large amounts of seeds are required for research, and (iv) new accessions are introduced in the genebank^13^. Genebank curators follow strict quality guidelines during the regeneration process to guarantee consistency and purity of the accessions across decades^13,18^. The quality management includes, among others, the scoring of morphological and agronomic traits for the phenotypic comparison with previous regenerations cycles. Since only 8.24% of the *Triticum aestivum* collection has been regenerated on average per year, the phenotypic data generated becomes non-orthogonal across years. In other words and considering an orthogonal data structure, the dataset looks like a two-dimensional array with phenotypic observations for 12,754 accessions in 70 years but with 92% of the data points corresponding to missing values. As a consequence, this particular data structure makes statistical analyses very challenging^12,14^. According to their growth habit we distinguish between 6,547 spring wheat accessions sown from February until April and 6,207 winter wheat accessions sown from September until December. The accessions were grown between 1946 and 2015 in Gatersleben, Germany (latitude 51°49′19.74′′N, longitude 11°17′11.80′′E, 110.5 m.a.s.l., black soil of clayey loam type, 9 °C average annual temperature, 490 mm average annual rainfall) in randomized plots with a size of up to 3.75 m².

**Fig. 1:**
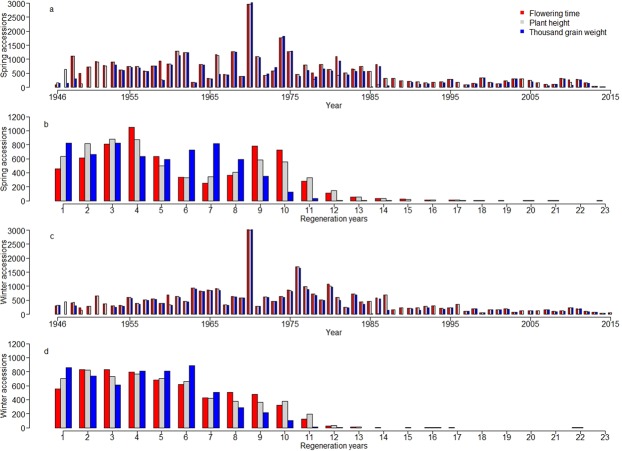
Non-orthogonal structure of historical phenotypic data gathered for 12,754 wheat accessions during 70 years of seed regeneration. (**a**) Number of regenerated spring wheat accessions per year with phenotypic observations for flowering time (FT), plant height (PH) and thousand grain weight (TGW) recorded between 1946 and 2015. (**b**) Frequency of regeneration years per accession with phenotypic observations for FT, PH and TGW for in total 6,547 different spring wheat accessions. (**c**) Number of regenerated winter wheat accessions per year with phenotypic observations for FT, PH and TGW gathered between 1946 and 2015. (**d**) Frequency of regeneration years per accession with phenotypic observations for FT, PH and TGW for in total 6,207 different winter wheat accessions.

The gathered data are non-orthogonal for the number of regenerated accessions per year, the number of regeneration years per accession as well as the number of traits recorded per year and accession (Fig. 1). For instance, an average number of 461 winter wheat accessions were evaluated for FT per year, but this quantity varied between 37 accessions in 2015 and 3,012 accessions in 1970 (Fig. 1c). A winter wheat accession was evaluated on average 5.12 times for FT, with the number of evaluation years per accession varying between one (555 accessions) and 22 (for a single particular accession) (Fig. 1d). While PH was evaluated for winter wheat in all 70 regeneration years, TGW records were only available for 58 years (Fig. 1c). A similar structure could also be observed for spring wheat.

Replicated checks were rarely used during seed regeneration or even differed within and across years. In total 0.21, 1.02 and 1.15% of the evaluated accessions had replicated records in at least one year for TGW, PH and FT, respectively. At one extreme for instance, the winter wheat accession “TRI 17296” has up to 11 additional records for PH in each of the 22 regeneration years from 1994 to 2015. In contrast the winter wheat accession “TRI 8404” has 22 additional records for PH in 1987 and was unreplicated in other regeneration years. Therefore, checks could not correct for any environmental source of variation in the field and could be ignored during data analysis.

Furthermore, historic events had an impact on the phenotyping intensity of genetic resources. For example, a protein screening on nearly all cereal genetic resources in 1970 caused the highest phenotyping intensity in spring and winter wheat (Fig. 1). Moreover, while until 1975 the seeds were stored at room temperature and had to be regenerated in 3–4 year intervals, the introduction of cold store facilities in 1976 extended these regeneration intervals to up to three decades. As a consequence, accessions acquired after the introduction of cold storage were less frequently phenotyped.

### Assessment of phenotypic traits

FT was measured when 50% of the plants of a plot reached the main flowering (growth stage 65 on the Zadok’s scale^19^). In the case of spring wheat, FT was expressed in days after the sowing date, while days after the 1^st^ of January of each year were considered as FT for winter wheat. PH was measured in cm from the soil surface to the top of the spike (including awns) at the begin of grain filling (growth stage on the Zadok’s scale 70^19^). TGW was measured in g after harvested seeds have been stored at room temperature and reached an overall grain moisture of ca. 12.5%. Until 2005, TGW was assessed based on the average weight of three samples, each containing 100 grains, which was then extrapolated to 1,000 grains. Since 2006 an automatic Marvin digital seed analyzer, GTA Sensors GmbH, Germany, considering a seed sample with up to 100 grains, was used to determine TGW.

Until 2011 the management of phenotypic data was manually at the genebank: First, the traits were recorded in field books, then transferred to card files and finally digitized for data storage and computational analysis. Since 2011 Personal Digital Assistants (PDAs) were used for data recording. For preparing this study, the digitized data were checked for correctness by random samples. Inconsistencies were double-checked against the original field books.

### Statistical model

The historical phenotypic data were analyzed separately for spring and winter wheat under the assumption of unreplicated, randomized plots and a missing-at-random missing value pattern^20^. The following linear mixed model was fitted to analyze the phenotypic data of each trait:

1 $${y}_{ij}=\mu +{g}_{i}+{a}_{j}+{e}_{ij},$$

where *y*_*ij*_ refers to the observed phenotypic value of the *i*^th^ accession in *j*^th^ year, *µ* is the population mean, *g*_*i*_ is the effect of the *i*^th^ accession, *a*_*j*_ refers to the effect of *j*^th^ year, and *e*_*ij*_ are the error terms associated to *y*_*ij*_. The error variances were assumed as specific for each year.

We first checked for suspicious years using a two-step outlier correction procedure. In the first step, year effects (*YE*) and the year specific error variance $({\sigma }_{{e}^{\ast }}^{2})$ in Eq. (1) were estimated by assuming accessions as random while years were considered fixed. Then, the coefficient of variation (*CV*) for each year was calculated as:

2 $$CV=\frac{\sqrt{{\sigma }_{{e}^{* }}^{2}}}{YE}.$$

If the *CV* of a specific year was deviating more than three standard deviations from the average *CV* of a particular trait, the quality of the records for the corresponding trait in that specific year was further inspected and potentially removed based on further criteria as described in the technical validation chapter. The second step corresponded to a record based outlier correction, in which Eq. (1) was fitted on the pre-corrected data obtained after the first step but this time assuming accessions as fixed and years as random. In order to test for outliers, we used the studentized residuals and applied a Bonferroni-Holm test to correct for multiple testing^21,22^. The outliers were again removed from the dataset. Finally, best linear unbiased estimations (BLUEs) for the accessions were estimated by fitting Eq. (1) on the outlier corrected data assuming accessions as fixed and years as random.

## Data Records

The relevant data of this study is available in the e!DAL-PGP-Repository^23^ and can be accessed here^24^. It includes three folders containing the original data (“Original_data_ISATab”), the outlier corrected data and BLUEs (“Processed_data”) as well as the corresponding software codes (“R_scripts”).

### The structure of the original historical data

In order to provide a unified and easy-to-read semantical description, the original data was formatted following the ISA-Tab format^25^. This includes one investigation file (“i_investigation.txt”), providing general project information, three study files describing the investigated spring and winter wheat accessions plus the weather data (“s_spring_wheat.txt”, “s_winter_wheat.txt” and “s_weather_data.txt”, respectively) as well as three assay files (“a_spring_wheat.txt”, “a_winter_wheat.txt” and “a_weather_data.txt”) containing the phenotypic records and weather parameters.

In detail, the study files for spring and winter wheat contain characteristics about: (i) the species (Organism), (ii) the accession identifier for users (Accession number), (iii) the unique database accession identifier (Accession ID), (iv) the sowing date in day.month.year format, (v) the harvest year and (iv) the country of origin as reported by collectors or donors (Origin country). The comment (Group) indicates countries, which were grouped together to create the overview presented in Table 1. As more accessions could be attributed to the former countries than to the current countries, they were grouped together with their former counterparts. These groups were Czechoslovakia, Yugoslavia and the Soviet Union. In the case of Germany, this group is unifying accessions of former East and West Germany. In the case of spring wheat, 6,547 accessions originate from 69 different provenances, with collection hotspots in India (11.82%), Austria (11.30%), and Iran (11.29%). In contrast, the 6,207 winter wheat accessions originate from 52 different provenances, with collection hotspots in Germany (11.00%), Italy (10.81%), Soviet Union (6.93%), and Iran (6.30%). The origins of 4.37% spring and 9.76% winter wheat accessions were unknown (Table 1). The study file for the weather data indicates the year and month in which weather parameters were recorded.

**Table 1:** Absolute quantities and relative proportion of the origins of 6,547 spring and 6,207 winter wheat accessions as reported by donors and collectors.

Spring wheat			Winter wheat		
Origin	Accessions	Percentage	Origin	Accessions	Percentage
India	774	11.82	Germany	683	11.00
Austria	740	11.30	Italy	671	10.81
Iran	739	11.29	Soviet Union	430	6.93
China	437	6.67	Iran	391	6.30
Libya	419	6.40	Czechoslovakia	340	5.48
Ethiopia	241	3.68	USA	332	5.35
Italy	221	3.38	Poland	310	4.99
Germany	211	3.22	France	271	4.37
Portugal	202	3.09	China	243	3.91
Soviet Union	190	2.90	Austria	218	3.51
Greece	189	2.89	Bulgaria	161	2.59
Afghanistan	173	2.64	Romania	159	2.56
USA	162	2.47	Greece	150	2.42
Mexico	140	2.14	Nepal	140	2.26
Nepal	104	1.59	Sweden	136	2.19
France	92	1.41	Pakistan	113	1.82
Turkey	89	1.36	Hungary	103	1.66
Spain	81	1.24	Yugoslavia	89	1.43
Kenya	77	1.18	India	80	1.29
Argentina	72	1.10	Afghanistan	75	1.21
Czechoslovakia	71	1.08	United Kingdom	70	1.13
Australia	67	1.02	Albania	66	1.06
Pakistan	65	0.99	Japan	65	1.05
Iraq	59	0.90	Netherlands	58	0.93
Japan	59	0.90	North Korea	47	0.76
Others (44)	587	8.97	Others (27)	200	3.22
Unknown	286	4.37	Unknown	606	9.76
Total	6547	100	Total	6207	100

Origins with minor proportions were grouped under Others with a total of 44 different origins for spring wheat and 27 for winter wheat.

The assay file for spring wheat provides 39,076, 36,905, and 29,676 phenotypic observations for FT, PH, and TGW corresponding to 6,535, 6,527, and 6,168 different accessions, respectively, collected throughout 69 different regeneration years (Table 2). Similarly, the assay file for winter wheat provides 31,817, 31,139, and 25,807 phenotypic observations for FT, PH, and TGW corresponding to 6,206, 6,164, and 5,841 different accessions, respectively, recorded in up to 70 different regeneration years. The assay file of the weather data provides monthly records of five weather parameters for 63 years from January 1953 to December 2015. Relying on this data, the average annual temperature was 9 °C with a minimum temperature of −30.6 °C measured in January 1963 and a maximum temperature of 39.8 °C measured in July 2015. The average annual rainfall was 493 mm with 1982 and 2007 being the driest (284.8 mm) and wettest (772.3 mm) year among all recorded years, respectively. The average air humidity (Moisture) was 79.6% varying from 61% in July 1976 to 93.8% in December 2012 and November 2014.

**Table 2:** Descriptive statistics of the original and outlier corrected historical data of spring and winter wheat for flowering time (FT), plant height (PH), and thousand grain weight (TGW).

Source	FT			PH			TGW		
	Original	Corrected	*Diff* (%)	Original	Corrected	*Diff* (%)	Original	Corrected	*Diff* (%)
**Spring wheat**									
*Records*	39,076	38,696	−0.97	36,905	36,854	−0.14	29,676	28,455	−4.11
*Accessions*	6,535	6,534	−0.02	6,527	6,527	0.00	6,168	6,168	0.00
*Years*	68	68	0.00	69	69	0.00	59	58	−1.69
${\sigma }_{G}^{2}$	14.24	14.49	1.75	250.71	253.09	0.95	29.11	29.97	2.94
${\sigma }_{Y}^{2}$	55.71	56.22	0.92	151.04	151.89	0.57	18.72	16.92	−9.66
${\sigma }_{e}^{2}$	14.64	10.77	−26.41	98.07	93.36	−4.80	18.16	16.18	−10.87
*h* ^2^	0.85	0.89	4.12	0.94	0.94	0.36	0.89	0.90	1.13
**Winter wheat**									
*Records*	31,817	31,566	−0.79	31,139	31,093	−0.15	25,807	25,114	−2.69
*Accessions*	6,206	6,201	−0.08	6,164	6,164	0.00	5,841	5,841	0.00
*Years*	69	69	0.00	70	70	0.00	58	57	−1.72
${\sigma }_{G}^{2}$	15.23	15.62	2.61	342.15	346.72	1.33	28.98	29.67	2.37
${\sigma }_{Y}^{2}$	72.36	71.95	−0.57	228.75	230.99	0.98	17.91	15.02	−16.16
${\sigma }_{e}^{2}$	9.08	6.48	−28.64	102.62	98.53	−3.99	16.13	13.76	−14.73
*h* ^2^	0.90	0.92	3.22	0.94	0.95	0.29	0.89	0.90	1.64

While *Records*, *Accessions* and *Years* indicate the number of phenotypic observations, the number of accessions and the number of observation years, respectively, ${\sigma }_{G}^{2}$, ${\sigma }_{Y}^{2}$, and ${\sigma }_{e}^{2}$ describe the genetic variance, the variance of the years and the error variance, correspondingly. The heritability of the traits is shown as *h²* and the impact of the outlier correction is shown as the relative difference (*Diff*) of the corrected data compared to the original data.

The software code “Outlier.correction.R” located in the folder “R_scripts” bases on the study and assay files and calculates the outlier corrected data for one example trait within spring and winter wheat.

### Ready-to-use processed historical data

The folder of the processed data contains the aggregated outlier corrected records for spring and winter wheat (“Outlier.corrected.spring.txt”, “Outlier.corrected.winter.txt”) which combine accession relevant information from the study and assay files for further analysis. After outlier detection for the spring wheat data, 0.97, 0.14, and 4.11% of the records were removed for FT, PH, and TGW, respectively (Table 2). In winter wheat 0.79, 0.15, and 2.69% outliers were removed for FT, PH, and TGW, respectively. This was accompanied by the loss of total FT information for one spring and five winter wheat accessions as well as the loss of one regeneration year for TGW for both, spring and winter wheat.

**Fig. 2:**
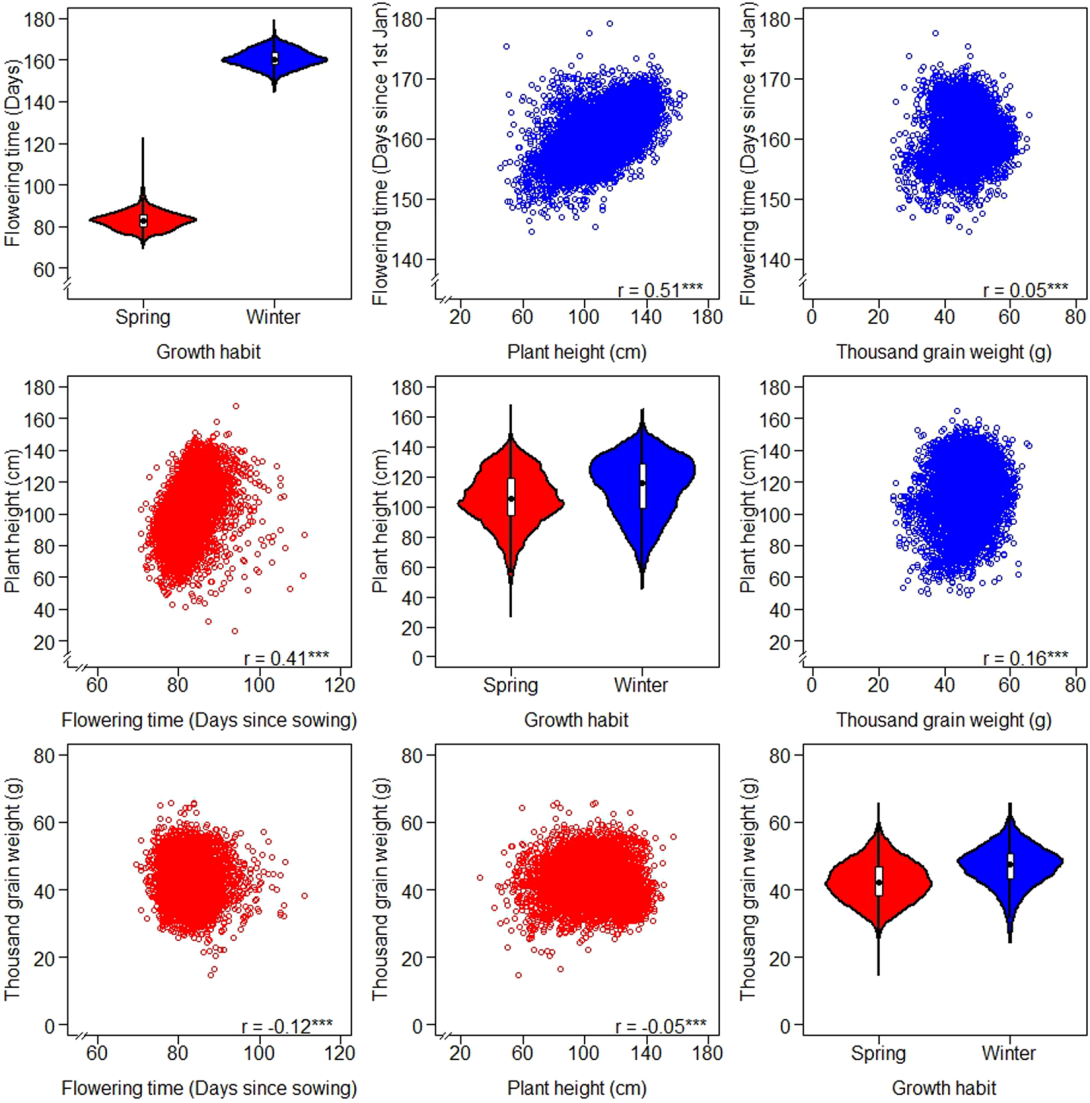
Matrix plot for the best linear unbiased estimations (BLUEs) for flowering time (FT), plant height (PH), and thousand grain weight (TGW) estimated for 6,547 spring (in red) and 6,207 winter wheat accessions (in blue). The diagonal shows the dispersion for each trait in spring and winter wheat as violin plot, where the black dot indicates the median value and the white box the flanking quartiles. The upper and lower triangles show the correlations among the three traits for spring and winter wheat, respectively. The Pearson’s correlation coefficients (r) differ significantly from zero (P-value < 0.001) as indicated by ***.

The software code “Estimation.of.BLUEs.R” bases on the outlier corrected data and calculates the BLUEs for one example trait. In the folder of the processed data the aggregated BLUEs for the traits within spring and winter wheat were stored as “BLUEs.spring.txt” and “BLUEs.winter.txt”. The spring wheat accessions flowered on average 82.97 days after sowing, with the earliest and latest accession flowering 69.57 and 122.62 days after sowing, respectively. In the case of winter wheat, accessions flowered on average at the 10^th^ of June, with the earliest and latest accession flowering at the 24^th^ of May and 28^th^ of June, respectively. The average PH for spring and winter wheat was 105.22 and 113.00 cm, correspondingly, with PH ranges of 26.63 to 167.49 and 45.27 to 164.50 cm, respectively. The average TGW for spring and winter wheat was 42.47 and 46.86 g, correspondingly, while TGW values fluctuated between 14.84 and 65.82 g for spring wheat and between 24.31 and 65.70 g for winter wheat. Moderate, but significant correlations (P-value < 0.001) between FT and PH were observed for spring (0.41) and winter wheat (0.51) as illustrated in Fig. 2.

## Technical Validation

### Heritability estimates indicate high data quality

The quality of the original historical data was assessed by estimating the heritability. In order to estimate the variance components, Equation 1 was fitted assuming both accessions and year as random effects. The heritability was calculated as:

3 $${h}^{2}=\frac{{\sigma }_{G}^{2}}{{\sigma }_{G}^{2}+\frac{{\sigma }_{e}^{2}}{Year}},$$

where ${\sigma }_{G}^{2}$ refers to the genetic variance of the accessions, ${\sigma }_{e}^{2}$ to the average error variance across regeneration years and *Year* to the average number of years each accession was evaluated. Heritability estimates between 0.85 (FT in spring wheat) and 0.94 (PH in spring and winter wheat) reflect the high quality of the original data (Table 2).

### Outlier correction boosted data quality

The outlier correction as described in the Methods section improved the heritability by up to 4.12% (FT in spring wheat) as compared to the original data (Table 2). In this particular case, the boost in heritability of FT in spring wheat was mainly explained by a 28.64% reduction of the error variance. However, removing too much outlier can be accompanied by the loss of valuable information and statistical power. For example, 4.11% of the data records for TGW in spring wheat were removed in total by the outlier correction. While 3.82% were discarded by the *CV*-based removal of the outlier year 1961, only 0.29% were removed by the subsequent Bonferroni-Holm test. Therefore, in order to minimize the data loss during the correction process, outlier years should only be discarded in well justified cases. This point is explained in the following section.

### Inspecting weather parameter helps to confirm outlier years

**Fig. 3:**
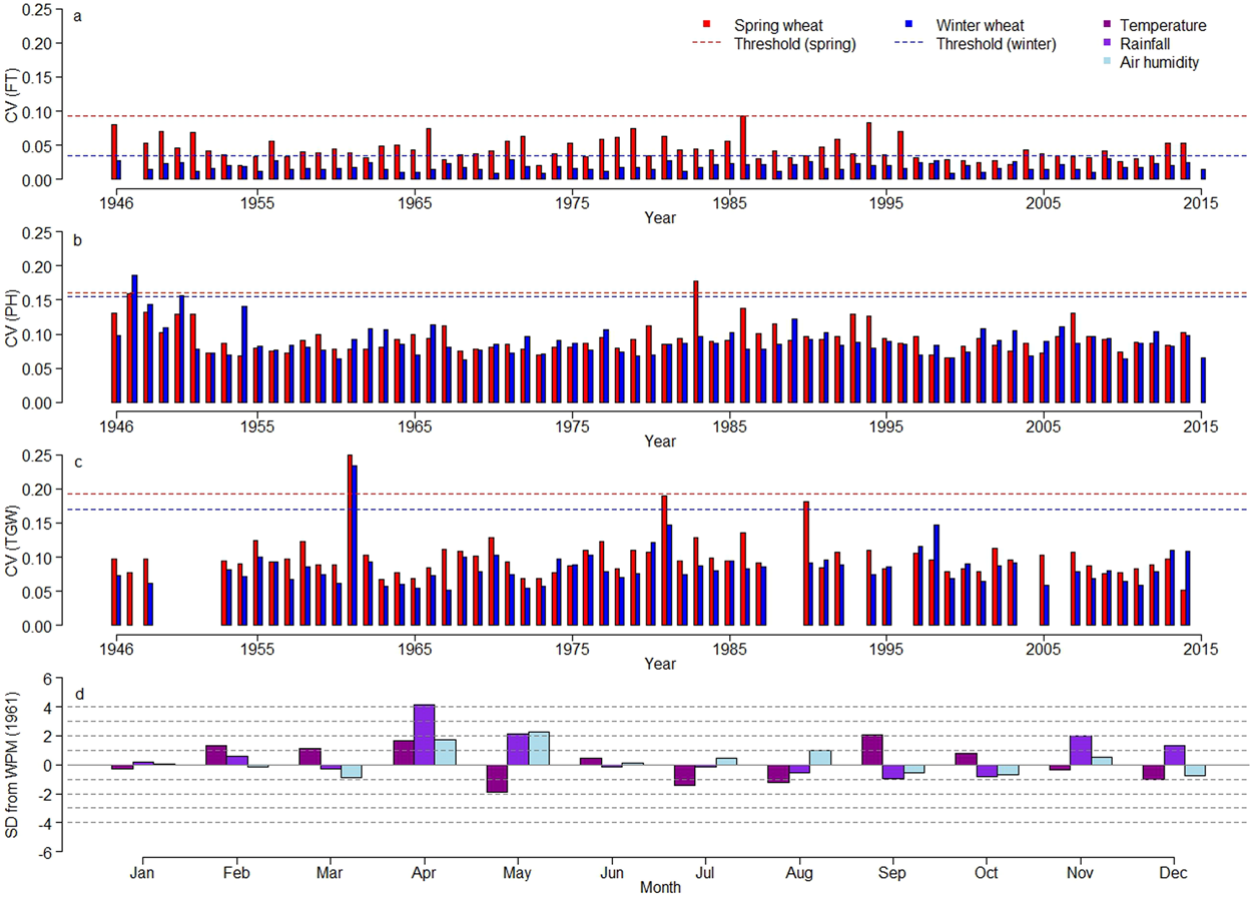
Identification of outlier years between 1946 and 2015 based on the coefficient of variation (*CV*) and their validation with weather parameters. *CV*s of spring and winter wheat are indicated in red and blue, respectively, while dashed lines indicate pre-defined threshold for outlier year candidates for: (**a**) flowering time (FT) (**b**) plant height (PH) and (**c**) thousand grain weight (TGW). Weather parameters of the outlier year 1961 for TGW are displayed in (**d**) and represented as the deviation (number of standard deviations, SD) from the normalized weather parameter mean (WPM) of the average monthly temperature (in magenta), the average monthly rainfall (in violet) and the average monthly air humidity (in light blue).

In general, higher *CV*s indicate lower year specific data quality (Fig. 3). *CV*s, which were deviating more than three standard deviations from the average *CV* across all years (under the assumption of normal distribution, 99.73% of the values cluster within an interval of the average value plus/minus three times the standard deviation) were inspected as potential outlier years. This threshold was clearly exceeded for TGW in 1961 for both spring and winter wheat (Fig. 3c). Figure 3d shows the standardized weather parameter as the deviation from the month specific average across years for T.avg, Rain and Moisture recorded from January to December of the year 1961. In April 1961, extraordinary high rainfall was observed, exceeding four times the standard deviation (Fig. 3d). This was accompanied by above-average T.avg and Moisture. In May 1961 Rain and Moisture still exceed the level of two standard deviations but the T.avg drops to a below-average level of nearly two standard deviations. This mixture of a warm, rainy and moist weather in April followed by cool rainy and moist conditions in May presumably provided best infection conditions for diseases like Septoria leaf blotch^26–28^. This disease spreads commonly by rain splash and could have negatively affected TGW^29,30^, leading to poor data quality of TGW in 1961. Thus, this particular year was excluded from the further analysis of TGW.

In the case of PH in winter wheat, *CV*s exceeded the threshold in 1947 and 1950, for which no weather data were available. Similarly, *CV* of PH in spring wheat during 1983 was also above the threshold, but in this case no weather anomalies could be observed (Fig. 3b). However, since removing these years from analysis had no effect on the heritability estimates, these years were kept in the dataset. For FT, the threshold was only exceeded for spring wheat in 1986 (Fig. 3a) but no weather anomalies were observed for that particular year. Nonetheless, a more detailed inspection revealed that a late sowing date (22^th^ to 24^th^ of April) during 1986 could have produced the drop in data quality for that particular year. Nevertheless, removing this year did not improved heritability estimates; thus, FT from 1986 was conserved in the outlier corrected dataset.

### Block-wise setup of plots during regeneration can influences precision of data analysis

A basic assumption in analyzing non-orthogonal data with the REML algorithm is that the missing data follows either a completely-at-random or a missing-at-random pattern^20^. In order to maintain germination ability and until the cold store facilities were introduced in 1976, each accession was regenerated on average every three to four years. This circumstance led often to a block-wise setup of regeneration plots for those accessions that simultaneously entered the genebank. In more detail, these blocks often correspond with collection hotspots and in consequence, potentially deviate from the missing-at-random assumption.

In order to quantify the bias in estimating first and second degree statistics, a resampling study was performed as described in a previous publication^12^. Briefly, a balanced sub-dataset for six years (1951, 1953, 1956, 1959, 1964 and 1970) was subtracted from the winter wheat data. This subset comprised 160 accessions originating from Germany (51), United States (42), Sweden (27), Greece (15), France (6), Afghanistan (6), Albania (6) and Great Britain (6). Three scenarios were simulated with (i) a block-wise missing structure with blocks composed by common origins, (ii) a block-wise missing structure with random composed blocks and (iii) a random missing structure ignoring block structure. Based on these missing data scenarios each accession was sampled for three out of six regeneration years and the BLUEs and variance components were estimated. Resampling was done 100 times for each scenario and the bias between the BLUEs from the resampled data and those of the orthogonal data was assessed. While no systematic bias in the estimation of BLUEs was observed, the standard deviations for the point estimates of the genetic variance in the block-wise missing scenarios were up to doubled compared to the random scenario. This last observation indicates a lower precision in estimating first and second degree statistics in block-wise regeneration structures. Therefore, an unstructured regeneration, as it is performed since the introduction of cold store facilities, is beneficial for the reuse of historical phenotypic data. Since the present historical data is a mixture of all simulated scenarios, we expect intermediate precisions in the estimation of first and second degree statistics.

### Orthogonal field trials confirm high data quality

The historical data of the winter wheat accessions were validated for FT and PH in orthogonal field trials as described in a previous publication^12^. Briefly, a random sample containing 36% of the winter wheat accessions was evaluated in replicated trials tested in up to 5 different environments following an alpha lattice design with a plot size of 0.4 m² and ~100 plants per plot. The BLUEs across environments for the validation trials were estimated and correlated to the historical data. On average the 2,236 accessions in the validation trials flowered 4.2 days earlier compared to their historical observations. Moreover, accessions tended to flower over time progressively earlier in the historical data, which was reflected in a rate of - 0.27 days per year. This rate is presumably a consequence of the also progressively increasing average spring temperatures (March, April and Mai) by a rate of 0.03 °C per year caused by global climate change. This temporal trend towards earlier flowering has been reported for several species^31–33^. In contrast, only a minor deviation was observed for PH, where the validated accessions were on average 3.6 cm higher compared to their historical observations. Nonetheless, the bias observed for FT could be mostly due to a linear shift, because Pearson’s correlation coefficients between the two datasets amounted to 0.84 for FT and 0.87 for PH; thus, confirming the high quality of the historical data.

## ISA-Tab metadata file

Download metadata file

## Data Availability

The linear mixed models were solved using the restricted maximum likelihood (REML) algorithm as implemented in ASReml-R^34^. All statistical analysis were implemented in R environment (Version 2.15.3)^35^. Example R-scripts for outlier correction (“Outlier.correction.R”) and estimation of BLUEs (“Estimation.of.BLUEs.R”) are included together with the data files^24^ in the e!DAL-Plant Genomics and Phenomics Research Data Repository (PGP)^23^. For applying the R-scripts it is necessary to download and save the data files and set the appropriate working directory in the scripts. The example script “Outlier.correction.R” runs for the analysis of TGW in winter wheat and produces the output files “Coefficient.of.variation.TGW.txt”, “Outliers.TGW.txt” and “Data.corrected.TGW.txt”, which correspond to the *CV* of the regeneration years, the removed outliers and the outlier corrected data, respectively. The script “Estimation.of.BLUEs.R” runs also for TGW in winter wheat based on the outlier corrected data and computes the BLUEs contained in the output “BLUEs.TGW.txt”. Both scripts can easily be adapted for the other traits and spring wheat following the annotations in scripts.
